# Reversible metamorphosis from Fe_3_O_4_ to FeO of epitaxial iron oxide films grown on the Fe-p(1 × 1)O surface

**DOI:** 10.1039/d0ra10650j

**Published:** 2021-03-19

**Authors:** M. Capra, A. Lodesani, A. Brambilla, M. Finazzi, L. Duò, F. Ciccacci, A. Picone

**Affiliations:** Department of Physics, Politecnico di Milano p.za Leonardo da Vinci 32 I-20133 Milano Italy andrea.picone@polimi.it

## Abstract

The reduction and oxidation of epitaxial Fe_3_O_4_ films grown by reactive deposition on a Fe-p(1 × 1)O surface have been investigated by means of Auger electron spectroscopy (AES), low energy electron diffraction (LEED) and scanning tunneling microcopy (STM). The as-grown iron oxide samples display a square LEED pattern with a lattice constant compatible with a p(1 × 1) bulk terminated Fe_3_O_4_(001) surface. STM topographic images of Fe_3_O_4_ are characterized by atomically flat terraces separated by highly oriented steps running along the (010) and (100) crystallographic directions of the substrate. Upon annealing at 800 K in an ultra-high vacuum, AES reveals that magnetite transforms to FeO. The sample exposes the (001) surface of the rock salt structure, with a lattice parameter close to that of bulk wüstite. The Fe_3_O_4_ phase can be recovered by oxidation at 10^−6^ mbar of molecular oxygen.

## Abstract 2

The reduction and oxidation of epitaxial Fe_3_O_4_ films grown by reactive deposition on a Fe-p(1 × 1)O surface have been investigated by means of surface science techniques.

## Graphical abstract

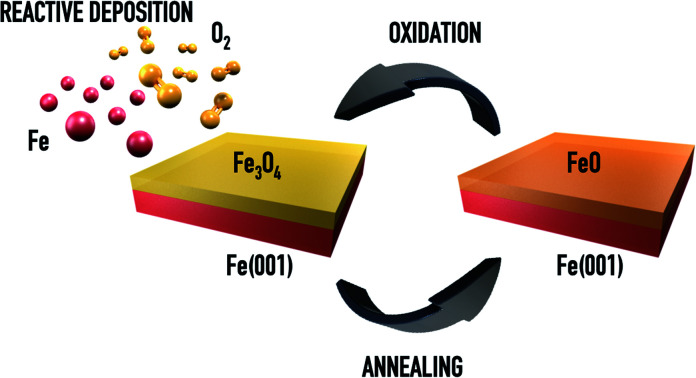

## Introduction

Iron oxides have been investigated for decades in several scientific disciplines, spanning physical chemistry^1–3^ to medicine.^4^ Owing to the different oxidation states assumed by Fe cations, Fe^2+^ or Fe^3+^, iron oxides can form various phases, with different stoichiometries and physical properties. Wüstite FeO is an antiferromagnetic insulator crystallizing in a rock salt lattice, where only Fe^2+^ cations are present. Magnetite Fe_3_O_4_ is a ferrimagnet containing a mixture of Fe^2+^ and Fe^3+^ ions, which assumes an inverse spinel structure. Above the Verwey transition temperature (*T*_v_ ∼ 120 K), Fe_3_O_4_ is a half metal, while below *T*_v_ it is insulating. Haematite (α-Fe_2_O_3_) adopts a corundum structure containing Fe^3+^ ions in octahedral sites. The stoichiometric α-Fe_2_O_3_, below 955 K, is an antiferromagnetic insulator.

The surface science paradigm, *i.e.* the preparation of well-defined model systems under highly controlled conditions, is the most appropriate to investigate the detailed atomic structure and chemical composition of oxide surfaces.^5–8^ Thin and ultra-thin Fe oxide films supported on metallic substrates like Pt,^9,10^ Ag,^11,12^ Fe,^13,14^, Ni,^15,16^ have been investigated by using this approach. After these investigations, it has been recognized that also new phases, with stoichiometries and physical properties deviating from those occurring in bulk samples, can be stabilized in epitaxial films with a thickness of few monolayers.^17^

The interconversion from one phase to another, depending on parameters like temperature and oxygen partial pressure, is particularly important, both from fundamental and applied points of view. In this frame, several recent papers describe the redox reactions occurring on iron oxide samples: Freindl *et al.* found that Fe_2_O_3_ films grown on Pt(111) can be reversibly reduced to Fe_3_O_4_ by annealing in vacuum,^18^ while Tang *et al.* reduced a bulk Fe_2_O_3_ sample to Fe_3_O_4_ by Ar ion sputtering followed by annealing.^19^ Jiang *et al.* investigated by means of ambient pressure scanning tunneling microscopy the effects induced by oxygen and carbon monoxide exposure on FeO(111) nano-islands grown on Au(111).^20^

The redox reactions occurring on Fe_*y*_O_*x*_ layers change drastically their physical and chemical properties, affecting the performances of the devices in which they are integrated. In heterogeneous catalysis, during the reaction the oxidation state of Fe oxide can be modified and influence the catalyst performances. For example, the oxidation of FeO films grown on Pt(111) induces the formation of a O–Fe–O trilayer, which is a key factor to activate the FeO/Pt(111) catalyst for low temperature CO oxidation.^21,22^ In exchange bias systems, where Fe is interfaced with an antiferromagnetic oxide, often the formation of Fe oxides is observed.^23–25^ The thermal treatments performed on the layered structure, such as the heating before the field cooling process, can induce redox reactions^26–28^ and affect the device properties. In magnetic tunnel junctions, often there is a thin Fe_*y*_O_*x*_ layer at the interface between the Fe electrodes and the MgO barrier, which can affect the device transport characteristics.^29^

In this paper we analyze the effect of annealing and oxygen exposure on an epitaxial Fe_3_O_4_ film grown on a Fe-p(1 × 1)O surface. The Fe-p(1 × 1)O sample is characterized by a single layer of oxygen atoms accommodated in the hollow sites of the Fe(001) surface and can be considered as a single layer of iron monoxide compressed by the underlying metal.^30–33^ The Fe-p(1 × 1)O passivated surface is more stable than the clean Fe(001) surface against the oxidation, thus it is an ideal template for the growth of Fe oxide in an oxygen atmosphere on a well-defined substrate.^5^ Our experiments show that the iron oxide films can be switched reversibly from Fe_3_O_4_ and FeO by cycles of high temperature annealing and exposure to molecular oxygen.

## Experimental details

The experiments have been performed in an ultra-high vacuum (UHV) chamber, at a base pressure of 10^−10^ mbar. The Fe(001) substrate was obtained by growing *in situ* a thick Fe film, about 500 nm, on a MgO(001) substrate. The Fe-p(1 × 1)O surface was obtained by following a well-established procedure, exposing the Fe(001) sample to 30 L of molecular oxygen (O_2_) and annealing in UHV to 800 K for 5 minutes.^5^ Fe_3_O_4_ films were grown by evaporating Fe in a 10^−6^ mbar O_2_ atmosphere, with the sample kept at 500 K during the deposition, as measured by a thermocouple positioned close to the sample. The Fe deposition rate was about 1 nm min^−1^, as evaluated by a quartz microbalance. The film thickness was estimated to be about 50 nm. The heating rate during the annealing was on average 1 K s^−1^, while during the cooling of the sample was about −2 K s^−1^. In the experiments where the reduction of Fe_3_O_4_ was induced, the films were annealed at 800 K in UHV with the same annealing and cooling rates used during the Fe_3_O_4_ growth. The scanning tunneling microscopy (STM) measurements were performed *in situ* by using an Omicron variable temperature STM. The images were acquired at room temperature in constant-current mode with tips obtained by electrochemical etching of W wires.

The Auger electron spectroscopy (AES) and low-energy electron diffraction (LEED) measurements performed by means of an Omicron SPECTALEED with a retarding field analyzer (total acceptance angle 102°). The primary energy of the electron beam for AES measurements was 3 keV.

## Results and discussion

**Fig. 1:**
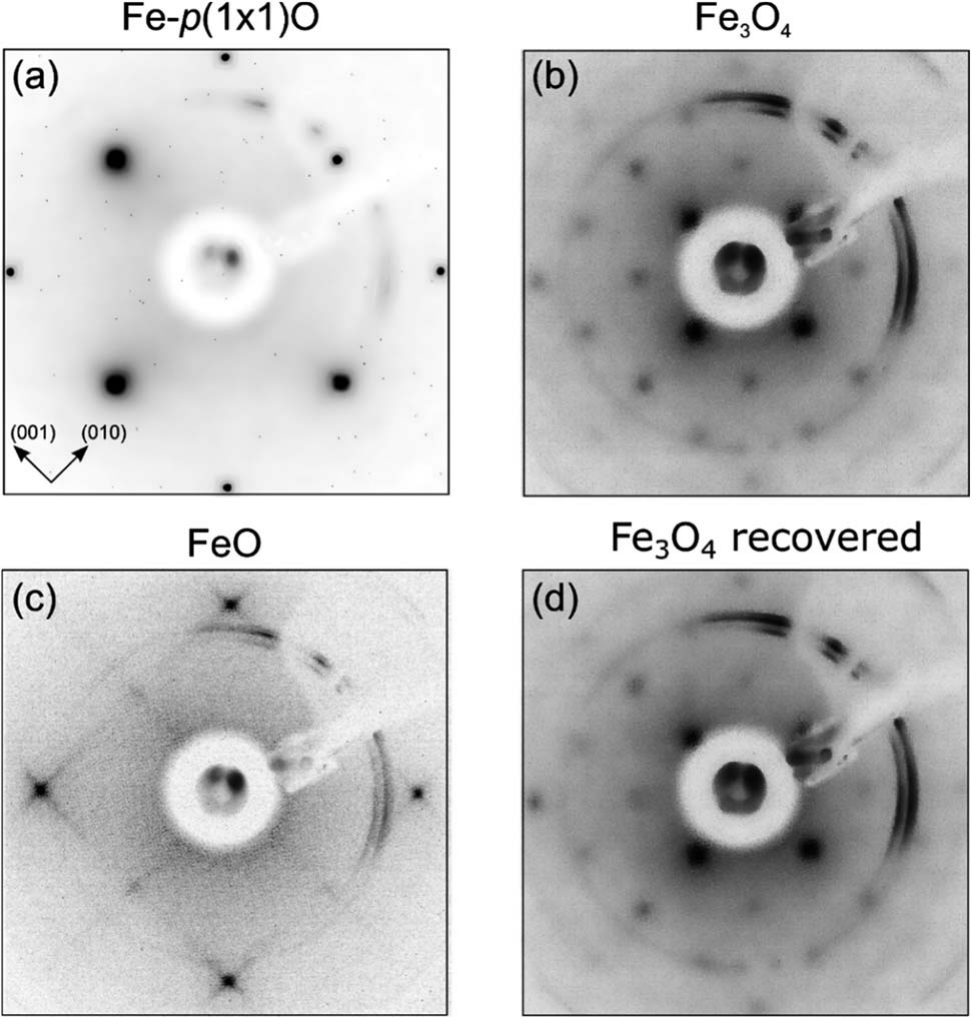
LEED images acquired on: (a) Fe-p(1 × 1)O substrate, (b) Fe_3_O_4_(001) surface obtained by reactive deposition of Fe in oxygen atmosphere, (c) FeO(001) surface after UHV annealing of Fe_3_O_4_, (d) Fe_3_O_4_(001) surface obtained by exposure of the FeO(001) sample to O_2_. Primary electron beam energy is 125 eV for each panel. The crystallographic directions are referred to the Fe(001) substrate.

Fig. 1 displays the LEED images acquired after each preparation step of iron oxide samples. Fig. 1(a) shows the diffraction pattern characteristic of the Fe-p(1 × 1)O surface, corresponding to a real space square lattice with a lattice parameter equal to *a*_p(1*×*1)_ = 2.86 Å. After the Fe deposition in O_2_ atmosphere, the LEED pattern in Fig. 1(b) indicates the presence of a p(2 × 2) reconstruction with respect to the Fe-p(1 × 1)O lattice. This pattern is attributed to the formation of a bulk-terminated Fe_3_O_4_(001) surface, which is characterized by a lattice parameter of about 0.59 nm. The stabilization of Fe_3_O_4_(001) is supported also by the AES and STM data presented in the following. The subsequent UHV annealing at 800 K lifts the p(2 × 2) phase and a square lattice similar to that of the Fe-p(1 × 1)O substrate appears in Fig. 1(c), although with different spot intensities and unit mesh dimensions. A quantitative evaluation of the ratio between the real space lattice parameter of the annealed sample (*a*_ann_) and that of Fe-p(1 × 1)O yields to 
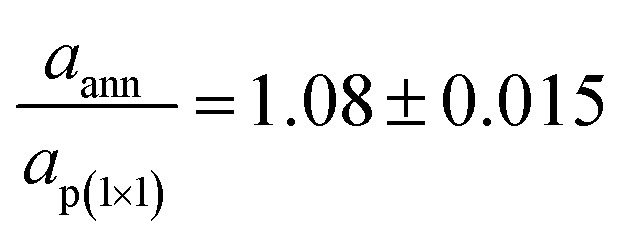
. This observation is consistent with the formation of a FeO(001) surface, for which the nominal lattice parameter of the primitive cell is about 3.07 A. The oxidation of the FeO(001) sample kept at 500 K restores the p(2 × 2) reconstruction [see Fig. 1(d)], attributed to the Fe_3_O_4_(001) surface.

It is worthwhile to mention that, for the Fe_3_O_4_(001) surface, a square unit mesh with a lattice constant of 0.843 nm is often reported, both in bulk magnetite samples^34^ and epitaxial films.^35^ This phase, which corresponds to a 
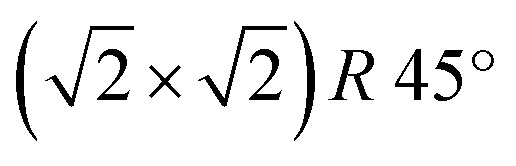
 reconstruction with respect to the bulk-terminated Fe_3_O_4_(001) surface, has been attributed to different causes, such as for instance oxygen vacancies in the topmost layer^35^ or electronic ordering.^34^ Recently, the commonly accepted model ascribes the 
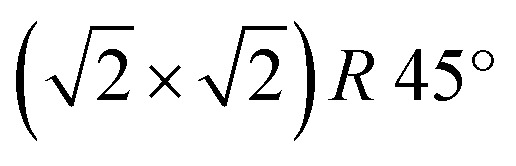
 phase to subsurface cation vacancies.^36^ In our case, we measure a surface lattice parameter of about 0.6 nm, which is consistent with an unreconstructed Fe_3_O_4_(001). The LEED images, acquired with a primary beam energy ranging from 50 eV to 200 eV in steps of 25 eV, do not display any extra spot due to the 
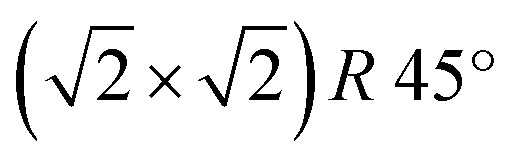
 reconstruction. Recently, the primitive Fe_3_O_4_(001) surface has been observed in experiments where the samples were kept at high temperature^37,38^ or after the deposition of atoms belonging to different species.^39^ In our case, the samples have been grown at 500 K in oxygen atmosphere, but the LEED and STM measurements have been performed at room temperature, therefore our results cannot be directly related to those of ref. 37 and ^38^. Further investigations are needed to rationalize the absence of the 
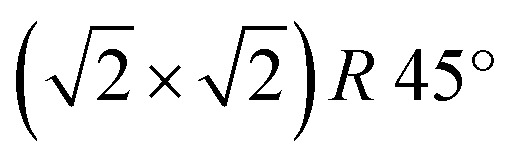
 phase.

**Fig. 2:**
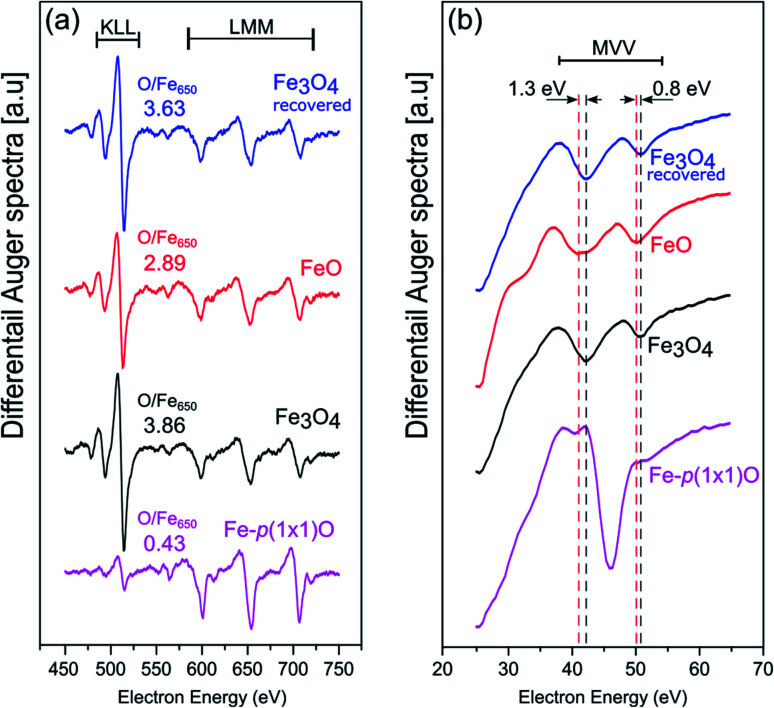
AES spectra acquired at (a) high and (b) low kinetic energy on the Fe-p(1 × 1)O substrate (purple spectra, bottom row), Fe_3_O_4_(001) surface obtained by reactive deposition of Fe on oxygen atmosphere (black, second row from bottom), FeO(001) surface after the UHV annealing of Fe_3_O_4_(001) (red, third row from bottom) and recovered Fe_3_O_4_(001) surface, obtained by oxidation of the FeO(001) sample (blue, top row). The dashed lines in panel (b) highlight the energy position of the Auger features related to FeO and Fe_3_O_4_.

Fig. 2(a) and (b) show the AES data acquired at high and low kinetic energies, respectively. The comparison of the intensities of the Fe LMM transitions with those of the O KLL peaks allows one to quantitatively estimate the chemical composition of the samples. On the Fe-p(1 × 1)O substrate, which is characterized by a single layer of oxygen atoms, the ratio between the O KLL peak and the Fe LMM peak at 650 eV is (O/Fe_650_)_p(1×1)_ = 0.43. After the reactive deposition of Fe in a O_2_ atmosphere, the ratio increases to (O/Fe_650_)_grown_ = 3.86, consistent with the formation of a thick iron oxide film. After UHV annealing, the ratio decreases to (O/Fe_650_)_ann_ = 2.89, suggesting a reduction of the film. The quantity 
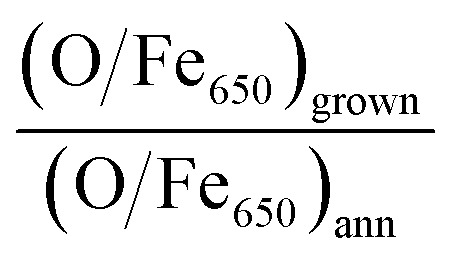
, which quantifies the relative stoichiometries of the as-grown and annealed films, provides a value of 1.33, in excellent agreement with that expected for a reduction from Fe_3_O_4_ to FeO. Finally, the O/Fe_650_ ratio increases again to a value of 3.63 after O_2_ exposure, pointing towards a recovery of the original magnetite film. The Auger peak at low kinetic energy reported in Fig. 2(b) corresponds to the MVV transition, which is particularly sensitive to the oxidation state of Fe atoms.^40,41^ The bottom spectrum of Fig. 2(b) acquired on the Fe-p(1 × 1)O is dominated by the peak of metallic Fe, with a minimum located at 46 eV. Additionally, another feature is visible at lower kinetic energy, partially superimposed onto the metal peak, which can be assigned to the presence of a single layer of iron oxide on the surface.^5^ The spectrum taken on the as-grown Fe_3_O_4_ is characterized by two features at about 42 eV and 51 eV. Upon the formation of FeO, these features shift at lower energies by 1.3 eV and 0.8 eV, respectively. Moreover, a small shoulder appears at about 35 eV. The spectrum acquired on the recovered Fe_3_O_4_ sample is identical to that of the as-grown magnetite.

**Fig. 3:**
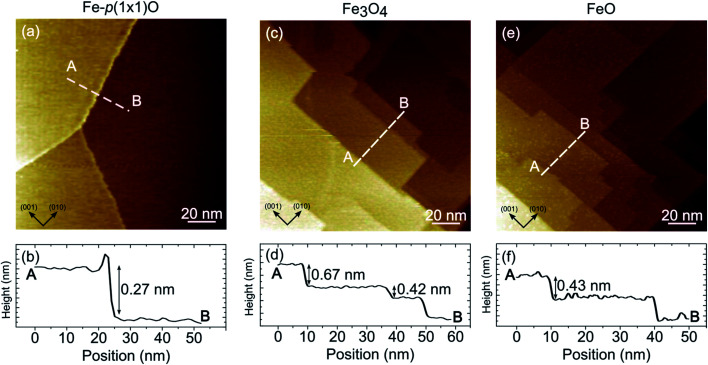
Scanning tunneling constant current images showing the topography of the Fe-p(1 × 1)O substrate (a), the Fe_3_O_4_(001) surface obtained by reactive deposition of Fe on oxygen atmosphere [panel (c)], FeO(001) sample after UHV annealing of Fe_3_O_4_(001) [panel (e)]. Panels (b), (d) and (f) display the topographic profiles corresponding to the dashed white lines of panels (a), (c) and (e), respectively. Image size is 140 × 140 nm^2^ for each panel. Tunneling parameters are *V* = 1 V, *I* = 1 nA for panels (a) and (c), *V* = 2.1 V, *I* = 0.5 nA for panel (e).

Fig. 3 illustrates the large-scale topography of the substrate [panel (a)], the as deposited Fe_3_O_4_ [panel (b)] and the FeO surface [panel (c)]. On the Fe-p(1 × 1)O sample, wide terraces separated by multiatomic steps are present, due to oxygen-induced bunching of Fe(001) steps.^31^ The step density along the (110) direction is about 0.7 × 10^−2^ nm^−1^. The line profile drawn in Fig. 3(a) crosses a bi-layer step [a monoatomic step in Fe(001) is 0.14 nm high], as visible in the topographic curve reported in Fig. 3(b). After the Fe_3_O_4_ growth, the surface morphology is characterized by a higher density of steps, about 2.5 × 10^−2^ nm^−1^ along the (110) direction, which are highly oriented along either the (001) or (010) crystallographic direction of the substrate. Panel (d) displays the line profile acquired across Fe_3_O_4_ terraces. Considering that the interlayer spacing between layers with the same termination is 0.21 nm, the measured topography corresponds to bunches of two and three steps of magnetite. The mesoscopic morphology of FeO reported in Fig. 3(e) is very similar to that of Fe_3_O_4_ in terms of steps density, orientation and height. The line profile drawn in Fig. 3(f) corresponds approximatively to a two-layer high step along the [001] direction of the rock-salt FeO [a monoatomic step in FeO(001) is 0.215 nm high].

It should be emphasized that, on the FeO samples, it was possible to obtain stable STM images only at high tip-sample voltage (above 2 V). Conversely, on Fe_3_O_4_ it was possible to measure also with small sample-tip bias, in agreement with the insulating and conductive nature of FeO(001) and Fe_3_O_4_(001) surfaces, respectively.

**Fig. 4:**
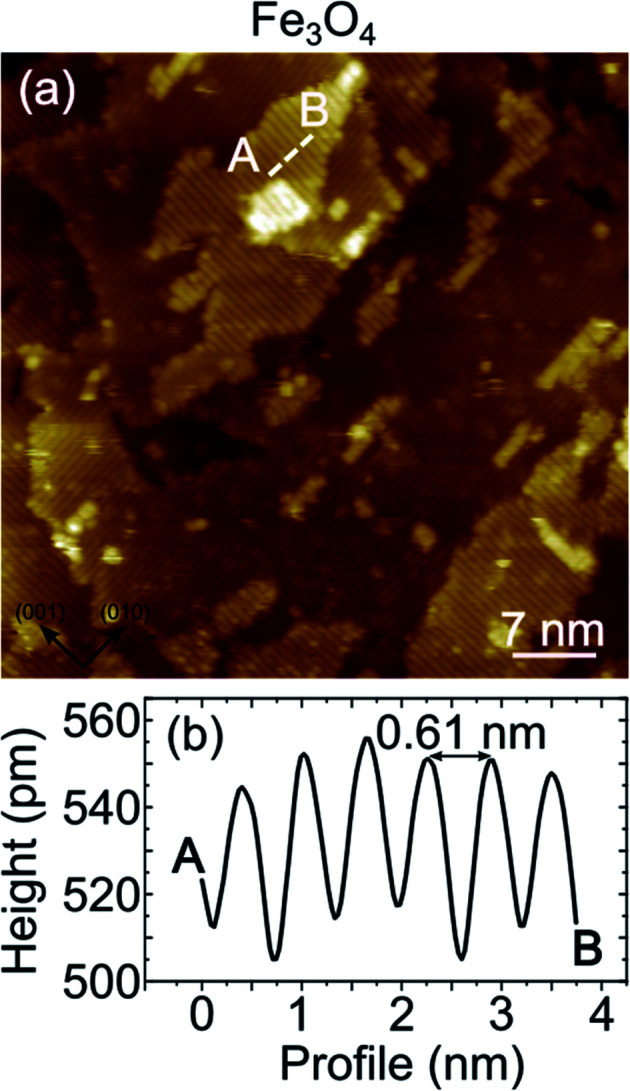
STM image at constant current showing the atomic corrugation of Fe_3_O_4_. Parallel lines running along the [010] and [001] directions of the Fe(001) substrate are visible, mutually orthogonal on terraces belonging to different layers. (b) Line profile displaying the atomic corrugation measured along the dotted line on panel (a). Image size is 50 × 50 nm^2^. Tunneling parameters are *V* = 0.1 V, *I* = 10 nA.

**Fig. 5:**
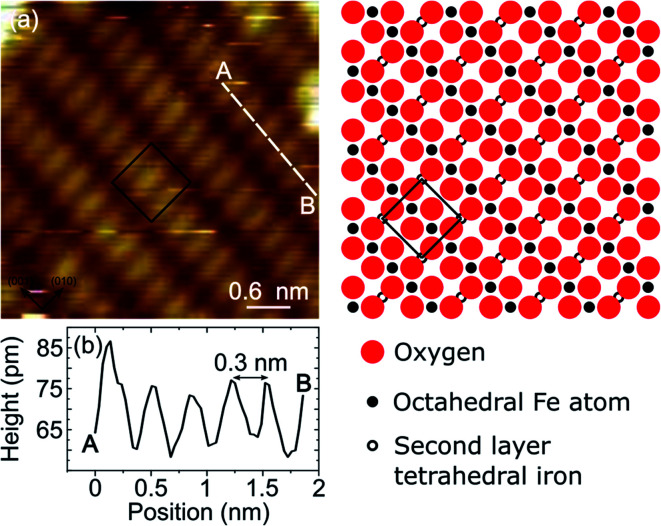
Panel (a) shows an atomically resolved STM constant current image of the Fe_3_O_4_(001) surface obtained by reactive deposition of Fe in oxygen atmosphere. Image size is 4 × 4 nm^2^, tunneling parameters are *V* = 0.1 V, *I* = 10 nA. Panel (b) displays the topographic profile corresponding to the dashed white line of panel (a), where an average atomic corrugation of about 12 pm is measured. On the right side a schematic model of the bulk-terminated Fe_3_O_4_(001) surface is reported. The black square indicates the surface unit cell.

On the Fe_3_O_4_, thanks to the good electrical conductivity of the surface, it was also possible to obtain atomically resolved images, shown in Fig. 4(a). The atomic corrugation is dominated by periodic rows with an apparent height of about 0.3 Å and separated by about 6 Å, as measured from the topographic line of Fig. 4(b). The rows belonging to different terraces are mutually rotated by 90°. These observations are similar to those reported for the reconstructed Fe_3_O_4_(001) surface of bulk sample and ultrathin films,^1^ despite we do not observe the
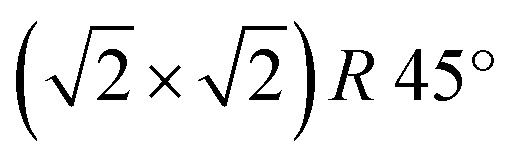
 superstructure in the LEED measurements, as already mentioned above. In Fig. 5 a highly resolved image is displayed, in which elongated features are present. In order to rationalize the structural data acquired by LEED and STM, the right panel of Fig. 5 reports a schematic model of the unreconstructed Fe_3_O_4_(001), which is characterized by a square unit cell compatible with the diffraction pattern measured by LEED. We suggest that, in our STM measurements, dimers of oxygen atoms separated by the octahedral Fe row are imaged as bright ovals, while the octahedral Fe atoms are imaged as depressions. We notice that our STM images of Fe_3_O_4_ differ noticeably from those reported in most of the literature, where the Fe cations are imaged as protrusions, despite there are reports in which anions are imaged as bright spots.^42^

In order to discuss the Fe_3_O_4_–FeO interconversion we recall that both phases are characterized by an oxygen face-centered-cubic lattice and differ only for the cation disposition. The Fe cations completely occupy the octahedral sites in FeO, while in magnetite there are Fe^3+^ in tetrahedral sites and a 50 : 50 mixture of Fe^3+^ and Fe^2+^ in octahedral sites. During the reduction of Fe_3_O_4_ to FeO oxygen atoms should be removed by the annealing and/or additional cations added to the iron oxide lattice. Generally, the UHV annealing of Fe_3_O_4_ bulk or Fe_3_O_4_ films grown on MgO modifies the surface structure but does not induce a complete reduction of magnetite to FeO,^35^ in agreement with the fact that even at a temperature of 1000 K and pressure of 10^−10^ mbar the free energies of Fe_3_O_4_ and FeO are −96.1 kJ mol^−1^ and −71.3 kJ mol^−1^, respectively.^43^ We suggest that in our case the transformation from Fe_3_O_4_ to FeO could be facilitated by additional Fe atoms provided by the Fe(001) substrate. The proximity of a reservoir of Fe atoms can modify the energetic balance, making FeO thermodynamically more stable than Fe_3_O_4_. A similar mechanism has been reported by Genuzio *et al.* in the case of Fe_2_O_3_ reduction to Fe_3_O_4_, where additional cations were supplied by Fe deposition on hematite.^44^

## Conclusions

Fe_3_O_4_(001) films have been grown by reactive molecular beam epitaxy on a Fe-p(1 × 1)O surface. The Fe_3_O_4_(001) LEED pattern forms a p(2 × 2) superstructure with respect to the substrate, revealing a surface structure compatible with a bulk terminated Fe_3_O_4_(001) sample. Highly resolved STM images have been obtained on magnetite, tentatively assigned to an imaging mode in which oxygen atoms are measured as protrusions. The Fe_3_O_4_(001) films can be converted in rock-salt FeO(001) by annealing the sample at 800 K in UHV. The FeO surface is characterized by a p(1 × 1) diffraction pattern with respect to the substrate, with a lattice constant similar to that of bulk Wüstite. The Fe_3_O_4_(001) and FeO(001) phases are clearly discernible also by AES, both in the high and low kinetic energy transitions. Finally, it has been shown that the Fe_3_O_4_(001) phase can be restored by the oxidation of FeO(001) at high temperature.

## Conflicts of interest

There are no conflicts to declare.

## Supplementary Material

## References

[cit1] Parkinson G. S. (2016). Iron oxide surfaces. Surf. Sci. Rep..

[cit2] Kim Y. J., Westphal C., Ynzunza R. X., Wang Z., Galloway H. C., Salmeron M., Van Hove M. A., Fadley C. S. (1998). The growth of iron oxide films on Pt(111): a combined XPD, STM, and LEED study. Surf. Sci..

[cit3] Kiejna A., Ossowski T., Pabisiak T. (2020). Gold nanostructures on iron oxide surfaces and their interaction with CO. J. Phys.: Condens. Matter.

[cit4] Zhang T., Xu Q., Huang T., Ling D., Gao J. (2020). New Insights into Biocompatible Iron Oxide Nanoparticles: A Potential Booster of Gene Delivery to Stem Cells. Small.

[cit5] Picone A., Riva M., Brambilla A., Calloni A., Bussetti G., Finazzi M., Ciccacci F., Duò L. (2016). Reactive Metal–Oxide Interfaces: a Microscopic View. Surf. Sci. Rep..

[cit6] Zhang J., Medlin J. W. (2018). Catalyst Design Using an Inverse Strategy: From Mechanistic Studies on Inverted Model Catalysts to Applications of Oxide-Coated Metal Nanoparticles. Surf. Sci. Rep..

[cit7] Netzer F. P. and Surnev S., Oxide Materials at the Two-Dimensional Limit, Springer, 2016, pp. 1–38

[cit8] Freund H.-J., Shaikhutdinov S., Nilius N. (2014). Model Studies on Heterogeneous Catalysts at the Atomic Scale. Top. Catal..

[cit9] Ranke W., Ritter M., Weiss W. (1999). Crystal structures and growth mechanism for ultrathin films of ionic compound materials: FeO(111) on Pt(111). Phys. Rev. B: Condens. Matter Mater. Phys..

[cit10] Davis E. M., Zhang K., Cui Y., Kuhlenbeck H., Shaikhutdinov S., Freund H. J. (2015). Growth of Fe_3_O_4_(001) thin films on Pt(100): Tuning surface termination with an Fe buffer layer. Surf. Sci..

[cit11] Lewandowski M., Pabisiak T., Michalak N., Miłosz Z., Babačić V., Wang Y., Hermanowicz M., Palotás K., Jurga S., Kiejna A. (2018). On the Structure of Ultrathin FeO Films on Ag(111). Nanomaterials.

[cit12] Waddill G. D., Ozturk O. (2005). Epitaxial growth of iron oxide films on Ag(111). Surf. Sci..

[cit13] Soldemo M., Vandichel M., Grönbeck H., Weissenrieder J. (2019). Initial Fe_3_O_4_(100) formation on Fe(100). J. Phys. Chem. C.

[cit14] Spiridis N., Barbasz J., Łodziana Z., Korecki J. (2006). Fe_3_O_4_(001) films on Fe(001): termination and reconstruction of iron-rich surfaces. Phys. Rev. B: Condens. Matter Mater. Phys..

[cit15] Lodesani A., Picone A., Brambilla A., Finazzi M., Duò L., Ciccacci F. (2020). 3-Dimensional nucleation of Fe oxide induced by a graphene buffer layer. J. Chem. Phys..

[cit16] Dedkov Y. S., Generalov A., Voloshina E., Fonin M. (2011). Structural and electronic properties of Fe_3_O_4_/graphene/Ni(111) junctions. Phys. Status Solidi RRL.

[cit17] Yang T., Song T., Callsen M., Zhou J., Chai J. W., Feng Y. P., Wang S. J., Yang M. (2019). Atomically Thin 2D Transition Metal Oxides: Structural Reconstruction, Interaction with Substrates, and Potential Applications. Adv. Mater. Interfaces.

[cit18] Freindl K., Wojas J., Kwiatek N., Korecki J., Spiridis N. (2020). Reversible oxidation–reduction
of epitaxial iron oxide films on Pt(111): magnetite–hematite interconversion. J. Chem. Phys..

[cit19] Tang Y., Qin H., Wu K., Guo Q., Guo J. (2013). The reduction and oxidation of Fe_2_O_3_(0001) surface investigated by scanning tunneling microscopy. Surf. Sci..

[cit20] Jiang Y., Zhu Y., Zhou D., Jiang Z., Si N., Stacchiola D., Niu T. (2020). Reversible oxidation and reduction of gold-supported iron oxide islands at room temperature. J. Chem. Phys..

[cit21] Sun Y.-N., Qin Z.-H., Lewandowski M., Carrasco E., Sterrer M., Shaikhutdinov S., Freund H.-J. (2009). Monolayer iron oxide film on platinum promotes low temperature CO oxidation. J. Catal..

[cit22] Sun Y.-N., Giordano L., Goniakowski J., Lewandowski M., Qin Z.-H., Noguera C., Shaikhutdinov S., Pacchioni G., Freund H.-J. (2010). The interplay between structure and CO oxidation catalysis on metal-supported ultrathin oxide films. Angew. Chem., Int. Ed..

[cit23] Młyńczak E., Luches P., Valeri S., Korecki J. (2013). NiO/Fe(001): magnetic anisotropy, exchange bias, and interface structure. J. Appl. Phys..

[cit24] Luches P., Bellini V., Colonna S., Di Giustino L., Manghi F., Valeri S., Boscherini F. (2006). Iron Oxidation, Interfacial Expansion, and Buckling at the Fe/NiO(001) Interface. Phys. Rev. Lett..

[cit25] Brambilla A., Picone A., Finazzi M., Duò L., Ciccacci F. (2011). Scanning tunneling microscopy investigation of CoO/Fe(001) and Fe/CoO/Fe(001) layered structures. Surf. Sci..

[cit26] Calloni A., Berti G., Brambilla A., Riva M., Picone A., Bussetti G., Finazzi M., Ciccacci F., Duò L. (2014). Electron spectroscopy investigation of the oxidation of ultra-thin films of Ni and Cr on Fe(001). J. Phys.: Condens. Matter.

[cit27] Riva M., Picone A., Bussetti G., Brambilla A., Calloni A., Berti G., Duò L., Ciccacci F., Finazzi M. (2014). Oxidation effects on ultrathin Ni and Cr films grown on Fe (001): a combined scanning tunneling microscopy and Auger electron spectroscopy study. Surf. Sci..

[cit28] Li X., Chang Y. C., Chen J. Y., Lin K. W., Desautels R. D., van Lierop J., Pong P. W. T. (2018). Annealing effect of NiO/Co_90_Fe_10_ thin films: from bilayer to nanocomposite. Phys. Lett. A.

[cit29] Kozioł-Rachwał A., Janus W., Szpytma M., Dróżdż P., Ślęzak M., Matlak K., Gajewska M., Ślęzak T., Korecki J. (2019). Interface engineering towards enhanced exchange interaction between Fe and FeO in Fe/MgO/FeO epitaxial heterostructures. Appl. Phys. Lett..

[cit30] Parihar S. S., Meyerheim H. L., Mohseni K., Ostanin S., Ernst A., Jedrecy N., Felici R., Kirschner J. (2010). Structure of O/Fe(001)-p(1 × 1) studied by surface X-ray diffraction. Phys. Rev. B: Condens. Matter Mater. Phys..

[cit31] Picone A., Brambilla A., Calloni A., Duò L., Finazzi M., Ciccacci F. (2011). Oxygen-induced effects on the morphology of the Fe(001) surface in out-of-equilibrium conditions. Phys. Rev. B: Condens. Matter Mater. Phys..

[cit32] Donati F., Sessi P., Achilli S., Li Bassi A., Passoni M., Casari C. S., Bottani C. E., Brambilla A., Picone A., Finazzi M. (2009). *et al.*, Scanning Tunneling Spectroscopy of the Fe(001)-p(1 × 1)O Surface. Phys. Rev. B: Condens. Matter Mater. Phys..

[cit33] Tange A., Gao C. L., Yavorsky B. Y., Maznichenko I. V., Etz C., Ernst A., Hergert W., Mertig I., Wulfhekel W., Kirschner J. (2010). Electronic Structure and Spin Polarization of the Fe(001)-p(1 × 1)O Surface. Phys. Rev. B: Condens. Matter Mater. Phys..

[cit34] Mariotto G., Murphy S., Shvets I. V. (2002). Charge ordering on the surface of Fe_3_O_4_(001). Phys. Rev. B: Condens. Matter Mater. Phys..

[cit35] Stanka B., Hebenstreit W., Diebold U., Chambers S. A. (2000). Surface reconstruction of Fe_3_O_4_(001). Surf. Sci..

[cit36] Bliem R., McDermott E., Ferstl P., Setvin M., Gamba O., Pavelec J., Schneider M. A., Schmid M., Diebold U., Blaha P., Hammer L., Parkinson G. S. (2014). Subsurface cation vacancy stabilization of the magnetite (001) surface. Science.

[cit37] Bartelt N. C., Nie S., Starodub E., Bernal-Villamil I., Gallego S., Vergara L., McCarty K. F., de la Figuera J. (2013). Order-disorder phase transition on the (100) surface of magnetite. Phys. Rev. B: Condens. Matter Mater. Phys..

[cit38] Arndt B., Lechner B. A. J., Bourgund A., Grånäs E., Creutzburg M., Krausert K., Hulva J., Parkinson G. S., Schmid M., Vonk V., Esch F., Stierle A. (2000). Order–disorder phase transition of the subsurface cation vacancy reconstruction on Fe_3_O_4_(001). Phys. Chem. Chem. Phys..

[cit39] Gargallo-Caballero R., Martin-Garcia L., Quesada A., Granados-Miralles C., Foerster M., Aballe L. (2016). *et al.*, Co on Fe_3_O_4_(001): towards precise control of surface properties. J. Chem. Phys..

[cit40] Sault A. G. (1994). Quantitative analysis of Auger lineshapes of oxidized iron. Appl. Surf. Sci..

[cit41] Riva M., Picone A., Bussetti G., Brambilla A., Calloni A., Berti G., Duò L., Ciccacci F., Finazzi M. (2014). Oxidation effects on ultrathin Ni and Cr films grown on Fe(001): a combined scanning tunneling microscopy and Auger electron spectroscopy study. Surf. Sci..

[cit42] Stoltz D., Önsten A., Karlsson U. O., Göthelid M. (2008). Scanning Tunneling Microscopy of Fe-and O-sublattices on Fe_3_O_4_(001). Ultramicroscopy.

[cit43] Ketteler G., Weiss W., Ranke W., Schlogl R. (2001). Bulk and surface phases of iron oxides in an oxygen and water atmosphere at low pressure. Phys. Chem. Chem. Phys..

[cit44] Genuzio F., Sala A., Schmidt T., Menzel D., Freund H.-J. (2014). Interconversion of α-Fe_2_O_3_ and Fe_3_O_4_ thin films: mechanisms, morphology, and evidence for unexpected substrate participation. J. Phys. Chem. C.

